# The effect of agroecosystem management on the distribution of C functional groups in soil organic matter: A review

**DOI:** 10.1007/s00374-021-01580-2

**Published:** 2021-07-01

**Authors:** Yuki Audette, Katelyn A. Congreves, Kimberley Schneider, Geovanna C. Zaro, Amanda L. P. Nunes, Hongjie Zhang, R. Paul Voroney

**Affiliations:** 1 School of Environmental Sciences, University of Guelph, 50 Stone Road East, Guelph, ON N1G 2W1 Canada; 2 Department of Plant Sciences, University of Saskatchewan, Saskatoon, SK S7N 5A8 Canada; 3 Department of Plant Agriculture, University of Guelph, Guelph, ON N1G 2W1 Canada; 4 Department of Agronomy, University Pitagoras Unopar, Arapongas, PR Brazil; 5 Lethbridge Research and Development Centre, Agriculture and Agri-Food Canada, Lethbridge, AB T1J 4B1 Canada

**Keywords:** Soil organic matter, Agricultural management, Functional groups, ^13^C NMR spectroscopy

## Abstract

To improve soil health and to aid in climate change mitigation, the quantity of soil organic matter (SOM) should be maintained or increased over the long run. In doing so, not only the total quantity of SOC but also the stability of SOC must be considered. Stability of SOC increases as a function of resistance to microbial decomposition or microbial substrate use efficiency through chemical, biological, and physical mechanisms including humification, hydrophobic moieties, molecular diversity, and formation of macroaggregates. One of the mechanisms that enhance stability confers changes in the distribution of C functional groups of SOM. To better understand and quantify how these changes are influenced by agricultural management practices, we collected 670 pairwise data from the body of literature that has evaluated changes in the distribution of C functional groups of SOM measured by solid-state ^13^C NMR spectroscopy. The types of agricultural managements discussed herein include (1) fertilization, (2) tillage, (3) crop rotation, (4) grazing, and (5) liming practices. Our meta-analyses show that these practices modify the distribution of C functional groups of SOM. Fertilization practices were associated with increased O-alkyl groups. Tillage resulted in increases in the SOC consisted of aromatic and carbonyl groups. Crop rotations, especially legume-based rotations, were found to increase the proportion of aromatic groups. Although there are fewer publications on tillage and crop rotation than on fertilization practices, the distribution of C functional groups may be more influenced by crop rotation and tillage practices than fertilization management—and should be a focus of future research.

**Supplementary Information:**

The online version contains supplementary material available at 10.1007/s00374-021-01580-2.

## Introduction

Soil organic matter (SOM) is a key element of soil health, as it affects the chemical, biological, and physical properties of soils. Characteristics of SOM are influenced by the two main controlling factors: (1) natural factors, such as climate, soil parent materials, land cover, and topography, and (2) human-induced factors, including land use, agricultural management, and degradation (Piccolo 2012). If both the natural and human-induced factors remain unchanged, SOM is believed to reach an equilibrium, reflecting a balance between C input and losses. However, as natural or human-induced forces fluctuate on the account of the changes in climate, land use, or agricultural management, SOM continues to cycle, resulting in gradual and selective changes in SOM and its properties over time (Ussiri and Johnson 2003).

Quantifying the characteristics of SOM is especially important after changes in agricultural management practices in order to understand the implications for C balance and soil health (Leifeld and Kögel-Knabner 2005). Currently, agriculture as a whole is considered a C source rather than a sink (Piccolo 2012). A decrease in SOC caused by intensive agricultural practices goes hand-in-hand with the deterioration of soil quality and functioning (Haynes et al. 1991; Shrestha et al. 2015)—which adversely affects the long-term sustainability of food production. By adopting targeted agricultural management practices designed to promote soil health, the SOC content should be maintained or increased over the long run (Kirkby et al. 2013). However, understanding how management influences SOC is not as simple as measuring the total quantity of SOC; the stability of SOC must also be considered. For C sequestration in soils, it is important to consider how to increase persistent SOM, which increases through various chemical, physical, and biological mechanisms, such as increasing (1) microbial substrate use efficiency (Coonan et al. 2020; Cotrufo et al. 2013), (2) humification (Stevenson 1994), (3) hydrophobic moieties (Piccolo et al. 2004), (4) molecular diversity (Lehmann et al. 2020), and (5) formation of macroaggregates (Kölbl and Kögel-Knabner 2004). In agricultural soils, SOC is mainly derived from crop residues (above- and below-ground) and animal manures (Pisani et al. 2016), which modify the activity of soil microbial communities thereby influencing the turnover of SOC (Ferrari et al. 2011). The accumulation and stability of SOC are often defined as its resistance to microbial decomposition (Leinweber et al. 2008) and are strongly affected by the composition of SOM—particularly the distribution of C functional groups of SOM (Piccolo 2012).

The stabilization of SOC is closely related to the conversion rate of each C functional group of SOM (He et al. 2018) including alkyl, O-alkyl, aromatic, and carbonyl groups (Table 1). Ono et al. (2011) reported that the highest proportion of organic C in plant litter, the forest floors, and the forest soils was observed in the O-alkyl groups and the decomposability of C was in the order of O-alkyl C > alkyl C > aromatic C > carbonyl C. O-alkyl groups influence the soil physical conditions, cation exchange reaction, anion retention, and biological activities (Haynes et al. 1991; Lima et al. 2009). It is considered that labile SOC decreases, while the stable SOC increases with soil depth (Soucémarianadin et al. 2018) and it has been observed that the proportions of O-alkyl groups decrease with soil depth, while the alkyl, carbonyl, and aromatic groups increase with increasing depth in the soil profile (Gao et al. 2021; Ussiri and Johnson 2003; Zhang et al. 2015). Although an increase in O-alkyl C content can be indicative of an accumulation of labile organic C (He et al. 2018), the positive correlation which is often observed between O-alkyl C conversion rate and soil clay content are likely explained by the underlying mechanism: clay enhances aggregate formation that reduces the organic C accessible to microbial decay (Kölbl and Kögel-Knabner 2004), thus stabilizing SOC.

**Table 1:** Chemical shift regions and their representative C functional groups in soil organic matter (SOM) extracted from soil and sediment samples measured by solid-state ^13^C NMR spectroscopy

Chemical shift (ppm)	Corresponding C group	Specific compounds	Comments	Reference
0–50	Alkyl groups: Alkyl C from cutin, suberin, lignin, lipids	31 ppm: Methylene groups (-CH_2_) in long aliphatic chains 16, 25, and 31 ppm: Non-substituted alkyl C	• Originally plant biopolymers • The metabolic products of soil microorganisms • Fatty acids are highly resistant to degradation and considered products through decomposition	He et al. 2018; Lima et al. 2009; Mylotte et al. 2016; Ono et al. 2011; Simpson and Preston, 2008; Smernik 2005; Stevenson 1994; Ussiri and Johnson 2003
50–110	O-alkyl groups: O-alkyl C and N-alkyl C from carbohydrates, peptides, and methoxyl C in lignin	55–56 ppm: Amino acid, protein and methoxy groups associated to lignin and lignin-like products 63 ppm: Carbohydrate 72–75 ppm: Polysaccharide rings and anomeric C in polysaccharides 103–104 ppm: Polysaccharide rings and anomeric C in polysaccharides 107 ppm: Anomeric C of carbohydrate	• The first component decomposed by microorganisms that eventually transform into aromatic or alkyl structures • Mono- and polysaccharides tend to adsorb onto the surface of clay minerals thereby enhancing soil aggregation	Fernandez et al. 2008; Haynes et al. 1991; He et al. 2018; Kögel-Knabner 2002; Lima et al. 2009; Mao et al. 2017; Ono et al. 2011; Ranatunga et al. 2017; Sierra et al. 2005; Simpson et al. 2008; Smernik 2005; Stevenson 1994; Ussiri and Johnson 2003; Zhang et al. 2019
110–165	Aromatic groups: Aromatic C including phenolic C from lignin	116 ppm: Guaiacyl units 129 ppm: Unsaturated C and protonated aryl-C 130 ppm: C-substituted aromatic C from lignin 150–152 ppm: Phenolic OH	• Generally increase with decomposition of organic matter by microbes • Condensed aromatic C groups are considered to be contributed from charred plant residues in soils due to natural vegetation fire • Aromatic groups derived from lignin is considered to be less degradable and accumulate in soils as partially decomposed SOC	Baldock et al. 1992; Dignac et al. 2017; Helfrich et al. 2006; Lima et al. 2009; Rumpel et al. 1998; Sierra et al. 2005; Simpson et al. 2008; Smernik 2005; Stevenson 1994; Ussiri and Johnson 2003
165–205	Carbonyl group: Carbonyl C and carboxyl C from fatty acids and peptides	168–169 ppm: Carboxylic groups of lipids 173 ppm: Amide and ester-C in lipids and proteins	• Slowly decomposed SOC, which decomposed more slowly than O-alkyl C but faster than aromatic C • Carboxyl and phenolic groups strongly complex with polyvalent cations, such as Al^3+^ and Fe^3+^ forming aggregate and insoluble conditions, thus the stability of aromatic groups could partially depend on the availability of polyvalent cations in soils	Calace et al. 2006; Lima et al. 2009; Posner 1966; Simpson et al. 2008; Smernik 2005; Stevenson 1994; Ussiri and Johnson 2003; Zhao et al. 2012

The ratio of alkyl to O-alkyl groups often is indicative of the degree of SOM decomposition, i.e., the degree of decomposition is high when the ratio is high (Leifeld et al. 2002; Zhao et al. 2012), while the total alkyl (i.e., the sum of alkyl and O-alkyl groups) to aromatic ratio is used as a sensitive index of the humification degree of SOM, i.e., the humification degree is high when the ratio is low (Zhang et al. 2019). The humified products are considered more stable with advancing humification degree (Zhao et al. 2021), which was enriched with aromatic groups and depleted in O-alkyl groups (Rodriguez et al. 2021). Phenolic groups as a part of aromatic groups are the two major functional groups known to complex with cationic nutrients (Klučáková and Kolajová 2014; Piccolo et al. 2019), thereby the sum of these two groups functionally represents SOM that is expected to enhance soil fertility.

**Fig. 1:**
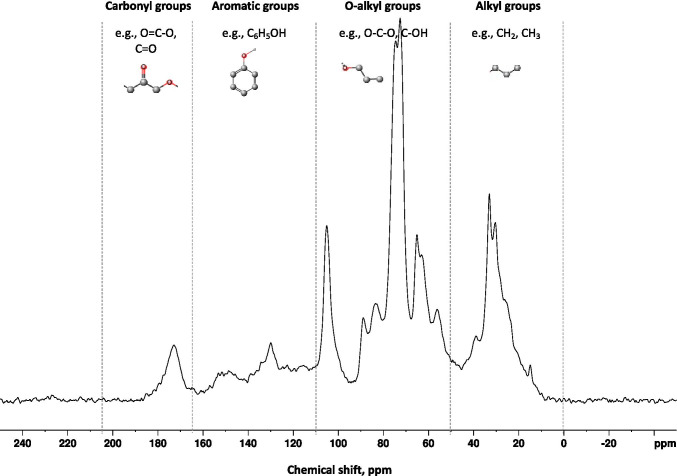
Solid-state ^13^C NMR spectra with chemical shift for the four C functional groups including alkyl, O-alkyl, aromatic, and carbonyl groups (a spectra for humin in source-separated organic compost from Audette et al. 2019)

The recent characterization and understanding of chemical properties of SOM is predominantly a result of advances in solid-state ^13^C nuclear magnetic resonance (NMR) spectroscopy and routine techniques of ^13^C cross polarization/magic angle spinning (Mao et al. 2008). Solid-state ^13^C NMR spectroscopy is a non-destructive powerful technique for characterizing SOM, which can differentiate broadly the four C functional groups (Fig. 1). Chemical shift of the four C functional groups and specific compounds belonging to each group observed in the various studies are summarized in Table 1. Most ^13^C spectra for SOM are broad and the assignment of either the specific functional groups or the specific compounds is difficult (Mao et al. 2017). For example, the regions of the chemical shift for O-alkyl and N-alkyl groups overlap; therefore, the N-alkyl groups are often included in O-alkyl groups (e.g., Kögel-Knabner 2002; Mao et al. 2017; Smernik 2005).

Although NMR spectra do not provide the structural organization of SOC at the molecular level and therefore is prone to losing details (Danchenko et al. 2020; Novotny et al. 2020) and often the region of these chemical shifts are not completely exclusive (Stevenson 1994), it still gives approximate distribution of C functional groups (Danchenko et al. 2020). By comparing in spectral regions, the degree of decomposition/humification of SOM in soils or the effects of different agricultural management practices on the distribution of C functional groups (Danchenko et al. 2020) could be determined with the solid-state ^13^C NMR spectroscopy (Helfrich et al 2006; Rodriguez et al. 2021).

Although the effects of agricultural managements on the simple quantification of SOC have been well studied, the effects on the distribution of C functional groups of SOM are not well understood. Therefore, in this review, we aim to investigate the effects of various agricultural management practices on the distrssibutions of C functional groups of SOM focusing on four major C functional groups, alkyl, O-alkyl, aromatic, and carbonyl groups measured by solid-state ^13^C NMR spectroscopy. Better understanding the role of agricultural management practices in regulating the stability and persistence of SOC will ultimately improve the capacity to adapt and mitigate climate change. Herein, we focus on five different agricultural management practices: (1) fertilization, (2) tillage, (3) crop rotation, (4) grazing, and (5) liming.

## Data collection and meta-analyses

The method of data collection and meta-analysis followed was that of Bai et al. (2018). Briefly, we performed literature review using web-based research engines including Web of Science and Google Scholar. Key search terms included soil organic matter, NMR spectroscopy, and various agricultural managements (i.e., fertilizers, manure, compost, grazing, tillage, crop rotation, and liming). Key elements of the selected studies were summarized in a Microsoft Excel database (details are found in the supplemental materials).

We collected a total of 670 pairwise data from 30 articles on the effect of agricultural management practices on the distribution of C functional groups measured by solid-state ^13^C NMR spectroscopy published from 1995 to 2020 worldwide, i.e., 373 pairwise data from 17 studies for fertilization practices, 222 pairwise data from 9 studies for tillage practices, and 75 pairwise data from 4 studies for crop rotation. This type of meta-analysis has known limitations due to relying on the available data, and the number of observations could be biased (Bai et al. 2018). In order to limit the influence of potential outliers, medians instead of means were used for visualizing the effects by each management (Bai et al. 2018). Few studies were found for the grazing and liming practices; therefore, we only performed the meta-analysis for the fertilization, tillage, and crop rotation practices. Biochar is often considered an organic fertilizer and has been recommended as a soil conditioner and a climate change mitigation strategy to increase SOC (Lehmann 2007; Woolf et al. 2010). However, the properties of biochar are highly dependent on the feedstock types and the production protocol used to produce the biochar (Xiao et al. 2018; Yuan et al. 2019); thus, biochar composition can be extremely variable (Jindo et al. 2020). We believe that further research is required prior to conducting a meta-analysis; therefore, biochar was excluded from the meta-analysis for fertilization.

A meta-analysis was conducted to quantify the effect of each agricultural management practice on the distribution of the C functional groups (i.e., Alkyl, O-alkyl, Aromatic, and Carbonyl groups), as well as the ratios of alkyl to O-alkyl (A/O), and alkyl to aromatic (A/Aroma) groups in soils measured by solid-state ^13^C cross polarization/magic angle spinning NMR spectroscopy. As previously mentioned in the “Introduction,” N-alkyl groups are included in O-alkyl groups due to their overlap peaks. For the effect of fertilization, the analysis was performed not only for soil samples, but also for SOM fractions (i.e., humic acids, fulvic acids, and particulate SOM fractions). Based on these data, we calculated the mean values of the effect of each management on the C functional groups and the ratios using the following equation (Eq. 1).

1 $$Effect=\frac{The\,proportion\,of\,functional\,group\,in\,the\,OM\,with\,management\,(\%)}{The\,proportion\,of\,functional\,group\,in\,the\,control\,(\%)}$$

When the values of effect are > 1, the management practice has a positive impact on the functional groups or ratio of interest; conversely, when the values of effect are < 1, the management practice has a negative impact compared to the control soil. Statistical analyses were performed using SAS version 9.4 (SAS Institute 2013). Medians, first quartile (*Q1*), third quartile (*Q3*), and standard deviation (*sd*) were computed by the Proc Means procedure, and least square means, standard error, and coefficient were computed by the Proc Mixed procedure. Tukey’s studentized range test was used for multiple comparisons of the effects of either temporal period (i.e., long-term repeated annual applications > 10 years, vs. short-term repeated annual applications < 10 years) for all practices or sample types for fertilizations. All data collected for the tillage practices and crop rotation were from soil samples. A type I error rate of *P* = 0.05 was used for all statistical tests. All data used in the study were the mean values where *n* > 3. Results of the statistical analyses are summarized in Tables S1 to S5 of the supplemental materials.

## Fertilization

The data collected to examine the effect of fertilization on the distribution of C functional groups are from studies that ranged in duration from 3 to 98 years. The temporal period of each management on each C functional group and the A/O and A/Aroma ratios by fertilizations were not significantly different (see Table S2 in the supplemental material); therefore, all data were used for further analysis. Although some recent studies showed that SOM fractions such as humic acids could be a good soil quality indicator representing SOM (e.g., Audette et al. 2021; Danchenko et al. 2020; Novotny et al. 2020; Savarese et al. 2021), significant differences between soil samples and SOM fractions were observed for alkyl groups and the ratio of A/O (see Table S3 in the supplemental materials); therefore, only the data from the studies that analyzed bulk soil samples (i.e., 227 pairwise data from 10 studies) were used for the further analysis.

**Fig. 2:**
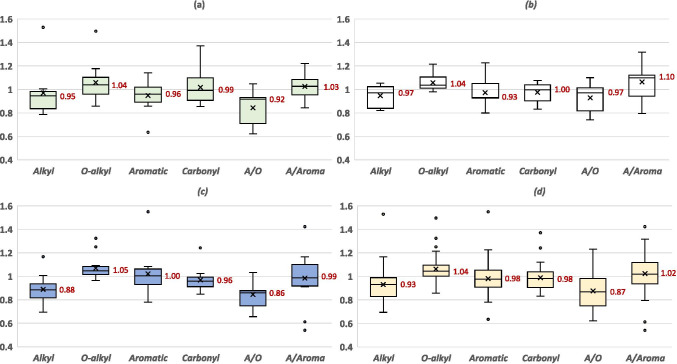
Spreads of the observations with effects of **a** organic fertilizers (*Organic*), **b** mineral balanced NPK fertilizers (*Mineral*), **c** combination of organic and mineral fertilizers (*Mixed*), and **d** total of all three fertilizers (*Total*) on alkyl, O-alkyl, aromatic, and carbonyl C groups, and the ratio of alkyl to O-alkyl groups (A/O), and the ratio of alkyl to aromatic groups (A/Aroma) in soil samples calculated from the 277 pairwise data collected 10 studies worldwide. No-fertilization of each study is considered the control (i.e., 1.0). The value shown in the box indicates median, the cross mark indicates mean, and lower and upper quartiles per management

Applications of fertilizers influence both quantity and the distribution of C functional groups of SOM; however, there is no consistent effect in the literature. For example, some organic fertilizers increase the SOC content compared to mineral fertilizers, such as sewage sludge and household organic compost, whereas others have no impact on the distribution of C functional groups, i.e., farmyard manure (Lima et al. 2009). Overall, we found that fertilizer application (i.e., any type of fertilizer, referred to as *Total*) positively affects the O-alkyl groups and the ratio of A/Aroma but negatively affects the ratio of A/O in soil samples (Fig. 2 and Table S1).

### Organic fertilizers

The most influential factors on the quality and quantity of SOM are thought to be the amount and type of C input by organic fertilizers in addition to plant residue. Organic fertilizers provide both plant-available nutrients and OM to soils; thus, they improve soil health by increasing the quantity of SOC. Plant residue and fresh organic fertilizers generally contain higher proportions of O-alkyl groups such as carbohydrates contributed from plant-derived polysaccharides (Ono et al. 2011), and lipids and proteins (i.e., N-alkyl groups) contributed from animal manures (Oades 1984).

Relatedly, our meta-analysis found that organic fertilizers, which include green waste, straw, sewage sludge, animal manures, and composts (i.e., used for our meta-analysis), positively affected the O-alkyl groups and the ratio of A/Aroma, while the alkyl and aromatic groups and the ratio of A/O were negatively affected (Fig. 2). Similarly, others have demonstrated that the application of organic fertilizers negatively affects the alkyl and aromatic groups, as well as SOC content, which is attributed to a priming effect promoting decomposition of native persistent SOC (Li et al. 2018; Zhang et al. 2015). Other studies show that higher proportions of N-alkyl groups including lignin, amino acids, peptides, and protein were observed in soils amended with either sewage sludge compost (Fernandez et al. 2008) or farmyard manure (Ferrari et al. 2011; He et al. 2018; Lima et al. 2009) compared to either unamended soils or soils amended with mineral fertilizers, both in short- (4 years) and long-term (> 40 years) studies. In addition to providing SOM containing a higher proportion of O-alkyl C, the positive effect on the O-alkyl groups may be explained by the fact that organic fertilizers may contain stable OM (i.e., mature compost) that protects labile SOC such as polysaccharides (i.e., O-alkyl) from biodegradation via repartition into the hydrophobic domains (Piccolo et al. 2004) thereby maintaining the proportion of O-alkyl groups.

Here, our data show that the effects of both maturity (i.e., composts vs. manure or sewage sludge) and source (i.e., manure vs. straw and green waste) of organic fertilizers on the distribution of C functional groups are not significantly different (see Tables S4 and S5 in the supplemental materials); however, both maturity and origin of organic fertilizers are considered to affect the quantity of SOC and stability of SOC differently. For example, Sauerbeck (1982) showed that accumulation of SOC in soils follows the order of green manure < straw < fresh manure < composted manure. Lipids and proteins contain hydrophilic moieties, which help to form macroaggregates by binding with soil mineral particles and microaggregates (Whalen and Chang 2002). Animal manures and composts generally contain a higher proportion of these compounds, thereby contributing to enhancing soil moisture content and nutrient turnover (Oades 1984), as well as enhancing stabilization of SOM by forming macroaggregates, ultimately improving soil structure. Further, the biological community is a key factor facilitating the link between the stability of SOC and the origin of the organic fertilizers (Li et al. 2018). For example, a strong correlation between the accumulation of O-alkyl groups and the *Acidbacteria* community composition was observed in soils amended with organic composts (Li et al. 2018). Animal manure and composts are relatively high in quality compared to crop straw due to their higher content of N, lower C/N ratios, and lower composition of phenol and lignin, which may accelerate decomposition of SOC (Luan et al. 2019). On the other hand, crop stover and straw are considered of lower quality and decompose C more slowly; as such, the O-alkyl C groups tend to increase in soils amended by these fertilizers (Chivenge et al. 2011).

### Mineral (or synthetic) NPK fertilizers

Our meta-analysis shows that mineral fertilizers also positively affect the O-alkyl groups and the A/Aroma ratio and negatively affect the alkyl and aromatic groups as well as A/O ratio compared to that of the control (Fig. 2). The increase in the O-alkyl groups due to the application of mineral fertilizers is most likely ascribed to greater quantities of crop residues. Although we did not find any significant effects on the proportion of aromatic groups (Fig. 2), several long-term studies found an enrichment of phenols/lignin monomers and alkyl-aromatics after > 10 years of applying NPK fertilizers (e.g., Leinweber et al. 2008; Li et al. 2015; Schjonning et al. 1994; Song et al. 2018). Others show that inorganic N fertilizers enhance the development of N-bonded aromatic groups (e.g., Chen et al. 2019; Gillespie et al. 2014; Moran et al. 2005) as a result of increased microbial activities (Song et al. 2018).

Unlike organic fertilizers, mineral fertilizers do not contain OM. Although some studies found no SOC accumulation with mineral fertilizer application, e.g., an 8 year study with annual mineral fertilizer applications (Luan et al. 2019), others have found differences (e.g., Moran et al. 2005; Randall et al. 1995; Song et al. 2018; Zhou et al. 2010). Mineral NPK fertilizers generally result in higher crop yields compared to the un-fertilized soils; therefore, the content of SOC may increase indirectly by addition of greater amounts of plant residue, especially root residues and exudates (Leinweber et al. 2008; Li et al. 2015).

As previously mentioned, the stabilization of SOC increases with increasing microbial substrate use efficiency. The availability of soil nutrients such as N and P, especially N, is known to correlate to microbial biomass and microbial use efficiency of plant litter (Cotrufo et al. 2013; Gillespie et al. 2014; Kirkby et al. 2013) and thereby is considered to be one of the regulatory factors influencing SOC degradation (Zhu et al. 2018). The addition of N fertilizers is also known to increase SOC persistence (Berg 2014; Moran et al. 2005) by altering soil microbial communities (Leff et al. 2015) and N-induced acidification can enhance the mineral-OM interaction in soils (Zhao et al. 2020). Despite our results, some studies show that the distribution of C functional groups generally shifts towards being more aromatic in nature when soils are amended with mineral fertilizers, whereas the application of fresh manure shifts SOC towards being more alkyl in nature (e.g., Song et al. 2018; Zhang et al. 2019). The C/N/P ratios strongly correlate with the stabilization of SOC (Abrar et al. 2021); thus, the long-term application of either organic or mineral fertilizers would either similarly affect or not affect the distribution of C functional groups of SOM depending on how the fertilization influences the C/N/P ratios in soil.

### Combination of organic and mineral fertilizers (mixed)

The application of organic fertilizers alone often results in lower crop yields than when supplemented with mineral fertilizers, whereas applying mineral fertilizers alone generally results in lower SOC contents than when applied with organic fertilizers (Wei et al. 2016). Thus, the mixed application of organic and mineral fertilizers is often considered a more practical approach for simultaneously enhancing crop yields and SOC (Wei et al. 2016). Similar to organic and mineral fertilizers, mixed fertilizers positively impact the O-alkyl groups, while the A/O ratio is negatively affected (Fig. 2). The effects of mixed fertilizers on the alkyl groups and the ratio of A/O are significantly different compared to organic or mineral fertilizations alone, i.e., the alkyl groups and the A/O ratio are negatively affected by the mixed fertilizers more than the other two (Fig. 2 and Table S3). Inconsistent observations for the effects of mixed fertilizers on the distribution of C functional groups were reported such as increasing alkyl (Zhang et al. 2019; Zhou et al. 2010), aromatic (Zhou et al. 2010), O-alkyl (He et al. 2018; Zhang et al. 2019), and carbonyl groups (Xu et al. 2017; Zhang et al. 2019) as well as the ratios of A/O and A/Aroma (Zhang et al. 2019), while decreasing alkyl (He et al. 2018), aromatic (Zhang et al. 2019), and O-alkyl groups (Xu et al. 2017) as well as the ratio of A/O (He et al. 2018). The impact on the distribution of C functional groups may highly depend on the origin and maturity of organic fertilizers as well as how the fertilization influences the C/N/P ratios in the soil.

## Tillage

The data collected to examine the effect of tillage practices on the distributions of C functional groups are from studies that ranged in duration from 8 to 56 years. Study duration was not found to have a significant impact on the distributions of C functional groups and ratios (see Table S2 in the supplemental material). Therefore, all data from the 9 studies conducted over a period of 8 to 56 years were used for further analysis.

Tillage practices are widely known to influence the biological, chemical, and physical properties of soil. The practice of no-till (NT) avoids any soil surface disturbance other than the disturbance due to planting (Bai et al. 2018) and this conservational practice is considered to increase soil biological activities, nutrient cycling, aggregate stability, and SOC content in the surface soil (Assunção et al. 2019; Bai et al. 2018; Hamza and Anderson 2005). Further, plants could use soil water more efficiently in soil under NT than under tillage practices due to lower water evaporation and higher water infiltration in the soil under NT (Phillips et al. 1980). Therefore, NT has been recommended for improving soil health, including the maintenance of SOC. On the other hand, recent studies show that occasional tillage practices after the addition of crop residue or animal manure either increase the rate of SOC storage (Gao et al. 2021; Mukumbuta et al. 2019) or have no effect on the quantity of SOC in the soil profile (up to 1 m) (Baker et al. 2007; Tang et al. 2019; Zhao et al. 2012) compared to the NT practice. Tillage may simply redistribute SOC from surface to deeper soil layers and could be beneficial for enhancing the stabilization of SOC (Baker et al. 2007; Mukumbuta and Hatano 2020). The study by Gao et al. (2021) showed that a 17-year tillage practice increased the microbial-derived SOC compared to the NT due to higher accumulation of microbial products in soil, which was contributed from increased microbial activities. Similarly, some studies show that NT preserves a less stable SOC than either reduced tillage or tillage practices (e.g., Murage and Voroney 2008; Panettieri et al. 2015). Further, Assunção et al. (2019) reported that NT favors emission of CO_2_ due to mineralization process and a higher accumulation of SOC in the surface under NT does not reflect the formation of stable SOC.

**Fig. 3:**
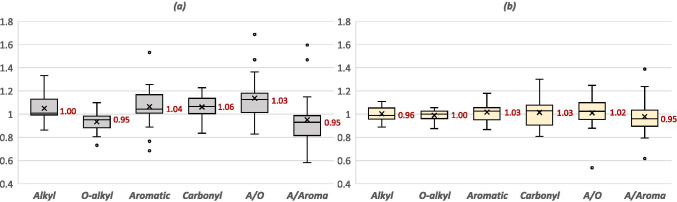
Spreads of the observations with effects of (**a**) conventional tillage and (**b**) reduced tillage practices on the functional C groups, including alkyl, O-alkyl, aromatic, and carbonyl groups, and the ratio of alkyl to O-alkyl groups (A/O), and the ratio of alkyl to aromatic groups (A/Aroma) calculated from the 222 pairwise data collected 9 studies worldwide, where no-tillage practice is considered a control (i.e., 1.0). The value shown in the box indicates median, the cross mark indicates mean, and lower and upper quartiles per management

Our results show that the effect of tillage practice on the C functional groups is more pronounced than that of fertilizer practices (see Table S1 in the supplemental material). Conventional tillage was found to positively affect the aromatic and carbonyl groups, as well as the A/O ratio, and negatively affected the O-alkyl groups and the A/Aroma ratio (Fig. 3). Similarly, reduced tillage practice increased the aromatic and carbonyl groups as well as the A/O ratio compared to NT; however, the effects are slightly less than those of conventional tillage (Fig. 2). Tillage facilitates the degradation of lipids, fatty acids, alkanes, and alkenes (Du et al. 2017; Laudicina et al. 2014; Shrestha et al. 2015), thereby providing more decomposed SOC structures consisting of aromatic groups including phenols, lignin monomers, and alkyl-aromatics structures (Assunção et al. 2019; Du et al. 2017; Gao et al. 2021; Laudicina et al. 2014; Shrestha et al. 2015). On the other hand, relative to tillage, the rate of decomposition may or has been shown to be lower under NT (Martins et al. 2011) resulting in the accumulation of more labile SOC (e.g., carbohydrates and O-alkyl C) (Gao et al. 2021). Therefore, the results suggest that tillage increases the proportion of more persistent C in soils compared with NT. However, the risk of loss of SOC from the soil surface as a result of erosion brought by water and wind could be higher under the conventional tillage depending on the topography (Gebhardt et al. 1985; Phillips et al. 1980). The influence of tillage on the distribution of C functional groups and potential implications in terms of C sequestration warrants further research.

## Crop rotation

Crop rotation, which is any system having more than two crops, is considered to be one of the practices promoting functional complexity of SOM (Lehmann et al. 2020) by stimulating diverse microbial populations (Lange et al. 2015) and rhizodeposits (Hemingway et al. 2019). Crop rotation is known to increase SOC content and crop yields (Bai et al. 2018; Gregorich et al. 2001).

**Fig. 4:**
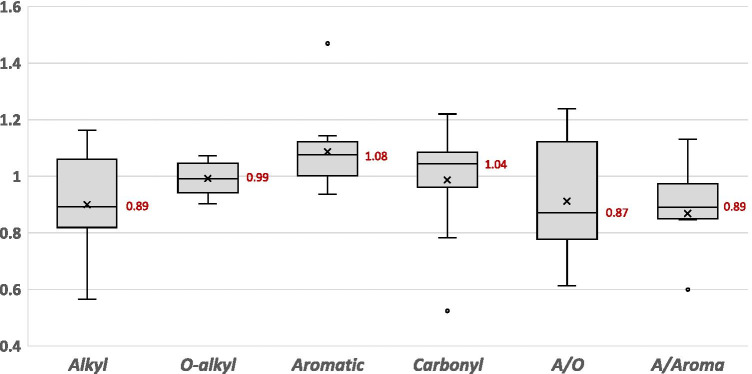
Spreads of the observations with effects of crop rotation on the C functional groups, including alkyl, O-alkyl, aromatic, and carbonyl groups, and the ratio of alkyl to O-alkyl groups (A/O), and the ratio of alkyl to aromatic groups (A/Aroma) calculated from the 69 pairwise data collected 3 studies worldwide, where monoculture system is considered a control (i.e., 1.0). The value shown in the box indicates median, the cross mark indicates mean, and lower and upper quartiles per management

The data collected to examine the effect of crop rotation on the distribution of C functional groups are from 4 studies that ranged in duration from 6 to 37 years. The length of time under crop rotation (6 years vs. > 10 years) was found to have a significant effect on the proportion of O-alkyl, aromatic, and carbonyl groups present (see Table S2 in the supplemental material), i.e., the long-term use of crop rotation increases the proportion of aromatic and carbonyl groups, while the O-alkyl groups are decreased compared with those studies employing crop rotation for 6 years. Therefore, the data from the single study conducted for 6 years were excluded from further analysis. The influence of crop rotation on the distribution of C functional groups is also more pronounced compared to the effect of fertilizer practice, despite fewer studies being available (see Table S1 in the supplemental materials). Crop rotation positively affected the aromatic and carbonyl groups and negatively affected the alkyl groups, the A/O, and A/Aroma ratios (Fig. 4). The mechanism explaining this effect is that crop rotations not only stimulate a diverse microbial community and rhizodeposits but also provide a higher amount of labile C consisting of O-alkyl C structures as plant biomass relative to a continuous monoculture (Arshad et al. 2011; Shrestha et al. 2015; Soon et al. 2007). This result is more pronounced in soils under legume-based rotations (Gregorich et al. 2001) where leguminous biomass is considered of high quality (i.e., a low C/N ratio) (Allard et al. 2005; Savarese et al. 2021). Further, Drinkwater et al. (1998) found that legume-based cropping systems with lower C/N ratios of OM supported the retention of SOC. In contrast, a higher proportion of O-alkyl groups and a lower proportion of aromatic groups were observed either in soils under continuous maize or continuous soybean (Qiao et al. 2018). In the soils under continuous barley, the SOC quality and quantity, alkyl C groups, and stability of aggregation were all negatively affected compared to the legume-based crop rotation (Arshad et al. 2011). Therefore, the distribution of C functional groups of SOM is influenced by having a crop rotation but by the specific crops included. We could only find 4 studies examining the effect of crop rotation on the distribution of the C functional groups of SOM despite how strong of an influence this practice may have; we believe more research on this topic is needed.

## Grazing

Grazing can have a highly detrimental or highly beneficial effect on C sequestration in soils (Bilotta et al. 2007; Zhou et al. 2016). In pasture systems, when the stocking rate is too high or plants are grazed too early, pasture productivity is often limited due to the inability of the plants to recover (Kenny et al. 2019; Rayburn and Sharpe 2019). Logically, this suppression of shoot and root growth can have serious negative effects on SOC stocks (e.g., see Naeth et al. 1991). On the contrary, grazing can stimulate plant growth and C inputs to below ground roots, increasing overall photosynthesis and pasture productivity (Kenny et al. 2019; Liebig et al. 2010; Rayburn and Sharpe 2019). By increasing photosynthesis, C inputs to the rhizosphere will also be increased as it is estimated that 30 to 40% of the photosynthates synthesized by the plant go directly to the plant roots (Kumar et al. 2006; Pausch and Kuzyakor 2017). Therefore, grazing management is likely to influence not only quantity, but also the stabilization of SOC. However, there have been few studies that have investigated the effects of grazing on the distribution of C functional groups of SOM using solid-state ^13^C NMR spectroscopy. In a study by Ganjegunte et al. (2005), the distribution of C functional groups in the humic and fulvic acids in soils was not significantly different under either continuous light (0.16–0.23 steers ha^−1^) and heavy grazing (0.56 steers ha^−1^) or no grazing for ~ 20 years; however, the O-alkyl groups were slightly greater in the humic acid fraction of the light grazing treatment compared to both the heavy grazing and grazing-free treatments. Several authors have also found that the more labile SOC fractions (i.e., light fraction C, microbial biomass C, and water-soluble OM) increase under grazing (e.g., Haynes 2000; Oduor et al. 2018; Ruis et al. 2017; Zani et al. 2020). This is explained due to increased cycling of plant material and the return of ingested plant material by grazing animals in the form of manure (Haynes 2000). Given the increasing recognition of the significant potential for agricultural grasslands to contribute to soil C sequestration (Conant et al. 2017; Teague et al. 2016), studies that integrate changes in SOC stocks with changes in the distribution of C functional groups of SOM as a result of grazing management are needed.

## Liming

Liming is a management practice applied on acidic soils to increase soil pH, which possibly changes mechanisms of flocculation, formation, and stabilization of macro- and microaggregates (Haynes and Naidu 1998). Liming often directly or indirectly influences both the quantity and the stabilization of SOC. Long-term liming (i.e., > 40 years) increases the quantity of persistent SOC due to the formation of organo-mineral fractions, especially with calcium ions, which act as a cation bridge between SOM and clay particles forming aggregates and lowering the C/N ratio in soils (Briedis et al. 2012; Fornara et al. 2011). In addition, increasing soil pH by liming provides favorable conditions for microbial growth and activity, as well as for N-mineralization and nitrification, thereby enhancing soil nitrate concentrations, microbial biomass C, and CO_2_ respiration rates (Fuentes et al. 2006). Liming strongly influences microbial community composition; for example, lower G + /G − bacteria ratios (Fornara et al. 2011) and a shift from fungi to bacteria (Wang et al. 2016) were observed in limed soils compared with un-limed soils. These observations are considered to be contributed from higher microbial metabolic quotients thereby enhancing microbial activity (Fornara et al. 2011) regulating decomposition and increasing persistent SOC consisting of alkyl structures with lower A/O ratio (Wang et al. 2016).

## Conclusions

The agricultural management practices studied herein influenced the proportions of alkyl, O-alkyl, aromatic, and carbonyl C functional groups of SOM. Fertilizer applications aimed at increasing crop yields increased the proportion of O-alkyl groups. Tillage was associated with more persistent SOC consisting of aromatic and carbonyl groups compared to soils under NT. Crop rotation, especially legume-based rotations, also increased the proportion of aromatic and carbonyl groups by increasing the molecular diversity of SOM—likely by way of improving the diversity of microbial diversity and rhizodeposits. Liming may improve the stability of SOC by providing a more favorable soil pH that supports microbial growth and activity.

Conservational practices, such as NT, have been recommended to sequester C in soils; however, our study points towards a higher degree of humified SOC in soils under tillage practices. Therefore, tillage appears to lead to more persistent SOC forms, compared to NT practices. As for the impact of crop rotation and tillage practice on the distribution of C functional groups of SOM, there are fewer publications on this topic than on fertilization practices; nevertheless, the distribution of C functional groups may be more influenced by crop rotation and tillage practices than fertilization management—and should be a focus of future research.

Other influencing factors in combination with agricultural management practices should be further explored. For example, soil types (Randall et al. 1995; Xu et al. 2017) or soil pH (Randall et al. 1995; Wang et al. 2016) could significantly influence the distribution of C functional groups of SOM in soils under different fertilizer regimes. Further, not only using advanced analytical techniques such as ^13^C NMR spectroscopy on soil samples but also studying how plant and other organic inputs may influence the distribution of C functional groups will further our understanding (Carteni et al. 2018).

It is well-known that the stability of SOC increases by various mechanisms including humification, molecular diversity, formation of macroaggregates, and increases in hydrophobic moieties. Here, we demonstrate how the stability of SOC is closely related to the distribution of C functional groups of SOM and impacted by different management practices. It is expected that this will contribute to better understanding the strategies towards sequestering more persistent C in soils and supporting soil health.

## Supplementary Information

Below is the link to the electronic supplementary material.

Supplementary file1 (DOCX 38.1 KB)

## Abbreviations

SOM: Soil organic matter
SOC: Soil organic carbon
NMR: Nuclear magnetic resonance
A/O: The ratio of alkyl to O-alkyl groups
A/Aroma: The ratio of alkyl to aromatic groups
NT: No-tillage
