# Supplementary material for: The effect of agroecosystem management on the distribution of C functional groups in soil organic matter: A review
Source: Biol Fertil Soils. 2021 Jul 1;57(7):881–94. doi: 10.1007/s00374-021-01580-2 (PMC8570350; doi:10.1007/s00374-021-01580-2)
Supplement: Supplementary file 1 — Supplementary file1 (DOCX 38.1 KB) [file 374_2021_1580_MOESM1_ESM.docx]

*Supplemental materials*

**The effect of agroecosystem management on the distribution of C functional groups in soil organic matter: A review**

Yuki Audette^1*^, Katelyn A. Congreves^2^, Kimberley Schneider^3^, Geovanna C. Zaro^1^, Amanda L. P. Nunes^4^, Hongjie Zhang^5^, and R. Paul Voroney^1^

^1^School of Environmental Sciences, University of Guelph, Guelph ON Canada N1G 2W1

^2^Department of Plant Sciences, University of Saskatchewan, Saskatoon SK Canada S7N 5A8

^3^Department of Plant Agriculture, University of Guelph, Guelph ON Canada N1G 2W1

^4^Department of Agronomy, University Pitagoras Unopar – Arapongas – PR, Brazil

^5^Lethbridge Research and Development Centre, Agriculture and Agri-Food Canada, AB Canada T1J 4B1

*Corresponding author, [yaudette@uoguelph.ca](mailto:yaudette@uoguelph.ca); 50 Stone Road East Guelph ON

**Table S1.** Effects of agricultural management practices (fertilization, tillage system or having a crop rotation) on various soil organic carbon functional groups (*i.e*., Alkyl, O-alkyl, Aromatic, Carbonyl groups) and the ratios of alkyl to O-alkyl (A/O) and alkyl to aromatic groups (A/Aroma) for soil organic matter compared to that of the control (*i.e.,* unfertilized, no tillage or mono-culture cropping system) based on the literatures published worldwide during 1995 - 2020. *Q1, Q3* and *sd* represent first quartile, third quartile and standard deviation, respectively.

| Management | | | Alkyl | O-alkyl | Aromatic | Carbonyl | A/O | A/Aroma |
| --- | --- | --- | --- | --- | --- | --- | --- | --- |
| Fertilizations (*n = 277*) | ***Organic*** | *Mean* | 0.97 | 1.06 | 0.95 | 1.02 | 0.84 | 1.03 |
|  |  | *Q1* | 0.84 | 0.96 | 0.89 | 0.91 | 0.71 | 0.95 |
|  |  | *Median* | 0.95 | 1.04 | 0.96 | 0.99 | 0.92 | 1.03 |
|  |  | *Q3* | 0.98 | 1.10 | 1.02 | 1.10 | 0.93 | 1.08 |
|  |  | *sd* | 0.21 | 0.18 | 0.13 | 0.14 | 0.15 | 0.11 |
|  | ***Mineral^†^*** | *Mean* | 0.95 | 1.06 | 0.97 | 0.98 | 0.93 | 1.056 |
|  |  | *Q1* | 0.84 | 1.01 | 0.92 | 0.90 | 0.82 | 0.94 |
|  |  | *Median* | 0.97 | 1.04 | 0.93 | 1.00 | 0.97 | 1.10 |
|  |  | *Q3* | 1.02 | 1.10 | 1.05 | 1.04 | 1.02 | 1.12 |
|  |  | *sd* | 0.09 | 0.07 | 0.13 | 0.08 | 0.13 | 0.17 |
|  | ***Mixed*** | *Mean* | 0.89 | 1.07 | 1.02 | 0.97 | 0.84 | 0.98 |
|  |  | *Q1* | 0.82 | 1.01 | 0.93 | 0.91 | 0.75 | 0.92 |
|  |  | *Median* | 0.88 | 1.05 | 1.00 | 0.96 | 0.86 | 0.99 |
|  |  | *Q3* | 0.94 | 1.08 | 1.06 | 0.99 | 0.88 | 1.10 |
|  |  | *sd* | 0.11 | 0.10 | 0.18 | 0.10 | 0.15 | 0.22 |
|  | ***Total*** | *Mean* | 0.93 | 1.06 | 0.98 | 0.99 | 0.88 | 1.02 |
|  |  | *Q1* | 0.83 | 1.00 | 0.91 | 0.90 | 0.75 | 0.99 |
|  |  | *Median* | 0.93 | 1.04 | 0.98 | 0.98 | 0.87 | 1.02 |
|  |  | *Q3* | 0.99 | 1.10 | 1.05 | 1.04 | 0.98 | 1.12 |
|  |  | *sd* | 0.14 | 0.12 | 0.15 | 0.11 | 0.14 | 0.18 |
| Tillage (*n = 222*) | ***Tillage*** | *Mean* | 1.05 | 0.93 | 1.06 | 1.06 | 1.14 | 0.95 |
|  |  | *Q1* | 0.99 | 0.88 | 1.01 | 1.00 | 1.01 | 0.82 |
|  |  | *Median* | 1.00 | 0.95 | 1.04 | 1.06 | 1.03 | 0.95 |
|  |  | *Q3* | 1.13 | 0.98 | 1.17 | 1.14 | 1.18 | 0.99 |
|  |  | *sd* | 0.11 | 0.08 | 0.17 | 0.11 | 0.19 | 0.23 |
|  | ***Reduced*** | *Mean* | 1.00 | 0.99 | 1.02 | 1.01 | 1.01 | 0.98 |
|  |  | *Q1* | 0.94 | 0.96 | 0.95 | 0.91 | 0.95 | 0.90 |
|  |  | *Median* | 0.96 | 1.00 | 1.03 | 1.03 | 1.02 | 0.95 |
|  |  | *Q3* | 1.05 | 1.03 | 1.06 | 1.08 | 1.11 | 1.03 |
|  |  | *sd* | 0.07 | 0.05 | 0.08 | 0.15 | 0.17 | 0.18 |
| Crop rotation^#^ (*n = 69)* | ***Rotation*** | *Mean* | 0.90 | 0.99 | 1.09 | 0.99 | 0.91 | 0.87 |
|  |  | *Q1* | 0.82 | 0.94 | 1.00 | 0.96 | 0.78 | 0.85 |
|  |  | *Median* | 0.89 | 0.99 | 1.08 | 1.04 | 0.87 | 0.89 |
|  |  | *Q3* | 1.06 | 1.05 | 1.12 | 1.08 | 1.12 | 0.97 |
|  |  | *sd* | 0.19 | 0.06 | 0.14 | 0.19 | 0.23 | 0.21 |

^†^Balanced NPK fertilizers only

^#^Crop rotation is anything with more than two crops

**Table S2.** Comparisons of the effects of agricultural management practices (fertilization, tillage system or having a crop rotation) on various functional C groups (*i.e*., Alkyl, O-alkyl, Aromatic, Carbonyl groups) and the ratios of alkyl to O-alkyl (A/O) and alkyl to aromatic (A/Aroma) groups compared to that of the control (*i.e.,* unfertilized, no tillage or mono-culture cropping system) between long-term repeated annual applications > 10 y (Long), *vs.* short-term (Short) repeated annual applications < 10 y. Numbers in parenthesis are standard errors.

|  | | | **Alkyl** | **O-alkyl** | **Aromatic** | **Carbonyl** | **A/O** | **A/Aroma** |
| --- | --- | --- | --- | --- | --- | --- | --- | --- |
| **Fertilization** | ***Organic*** | **Short** | 1.07*a*  (0.06) | 1.02*a*  (0.04) | 0.93*a*  (0.05) | 0.96*a*  (0.04) | 1.11*a*  (0.07) | 1.13*a*  (0.06) |
|  |  | **Long** | 1.01*a*  (0.04) | 1.03*a*  (0.03) | 0.96*a*  (0.04) | 1.01*a*  (0.03) | 0.93*a*  (0.05) | 1.02*a*  (0.05) |
|  | ***Mineral*** | **Short** | 0.96*a*  (0.07) | 1.01*a*  (0.05) | 1.01*a*  (0.06) | 1.01*a*  (0.05) | 0.96*a*  (0.08) | 1.00*a*  (0.08) |
|  |  | **Long** | 0.96*a*  (0.04) | 1.05*a*  (0.03) | 1.02*a*  (0.03) | 0.97*a*  (0.03) | 0.97*a*  (0.05) | 1.07*a*  (0.04) |
|  | ***Mixed*** | **Short** | 0.92*a*  (0.09) | 1.07*a*  (0.07) | 0.98*a*  (0.08) | 0.92*a*  (0.07) | 0.86*a*  (0.11) | 1.04*a*  (0.10) |
|  |  | **Long** | 0.96*a*  (0.04) | 1.03*a*  (0.03) | 1.02*a*  (0.03) | 1.03*a*  (0.03) | 0.95*a*  (0.04) | 0.99*a*  (0.04) |
|  | ***Total*** | **Short** | 0.98*a*  (0.04) | 1.03*a*  (0.03) | 0.97*a*  (0.04) | 0.96*a*  (0.03) | 0.98*a*  (0.05) | 1.06*a*  (0.05) |
|  |  | **Long** | 0.97*a*  (0.02) | 1.03*a*  (0.02) | 0.98*a*  (0.02) | 1.00*a*  (0.02) | 0.94*a*  (0.03) | 1.03*a*  (0.03) |
| **Tillage** | ***Tillage*** | **Short** | n/a | n/a | n/a | n/a | n/a | n/a |
|  |  | **Long** | 1.05  (0.03) | 0.93  (0.03) | 1.06  (0.03) | 1.06  (0.03) | 1.14  (0.03) | 0.95  (0.03) |
|  | ***Reduced tillage*** | **Short** | 0.95*a*  (0.07) | 1.02*a*  (0.07) | 1.00*a*  (0.07) | 1.01*a*  (0.07) | 0.91*a*  (0.07) | 0.98*a*  (0.07) |
|  |  | **Long** | 1.02*a*  (0.04) | 0.98*a*  (0.04) | 1.03*a*  (0.04) | 1.02*a*  (0.04) | 1.05*a*  (0.04) | 0.98*a*  (0.04) |
| **Crop rotation** | | **Short** | 0.95*a* (0.13) | 1.33*a*  (0.10) | 0.63*b*  (0.13) | 0.69*b*  (0.13) | 0.73*a*  (0.13) | 1.14*a*  (0.19) |
|  |  | **Long** | 0.90*a* (0.06) | 0.99*b*  (0.06) | 1.09*a*  (0.05) | 1.03*a*  (0.05) | 0.91*a*  (0.06) | 0.92*a*  (0.05) |

Means with the same letter indicate no significant difference within the same functional groups among practices according to Tukey’s multiple range test (*P = 0.05*). n/a = no data available.

**Table S3.** Comparisons of the effects of fertilization on various functional C groups (*i.e*., Alkyl, O-alkyl, Aromatic, Carbonyl groups) and the ratios of alkyl to O-alkyl (A/O) and alkyl to aromatic (A/Aroma) groups compared to that of the control (*i.e.,* unfertilized system) between soil samples and SOM fractions (SOM, humic acids, fulvic acids and particulate SOM). Numbers in parenthesis are standard errors.

|  | | | **Alkyl** | **O-alkyl** | **Aromatic** | **Carbonyl** | **A/O** | **A/Aroma** |
| --- | --- | --- | --- | --- | --- | --- | --- | --- |
| **Fertilization** | ***Organic*** | **SOM** | 1.08*a*  (0.05) | 0.99*a*  (0.03) | 0.95*a*  (0.04) | 0.97*a*  (0.04) | 1.14*a*  (0.04) | 1.09*a*  (0.05) |
|  |  | **Soil** | 0.97*ab*  (0.04) | 1.06*a*  (0.03) | 0.95*a*  (0.04) | 1.02*a*  (0.03) | 0.84*bc*  (0.05) | 1.02*a*  (0.05) |
|  | ***Mineral*** | **SOM** | 0.98*ab*  (0.05) | 1.01*a*  (0.04) | 0.99*a*  (0.05) | 0.98*a*  (0.04) | 1.06*ab*  (0.06) | 1.03*a*  (0.06) |
|  |  | **Soil** | 0.94*ab*  (0.04) | 1.06*a*  (0.03) | 0.97*a*  (0.04) | 0.98*a*  (0.03) | 0.93*bc*  (0.04) | 1.07*a*  (0.05) |
|  | ***Mixed*** | **SOM** | 1.08*ab*  (0.05) | 0.95*a*  (0.04) | 1.00*a*  (0.05) | 1.09*a*  (0.04) | 1.14*a*  (0.06) | 1.03*a*  (0.06) |
|  |  | **Soil** | 0.89*b*  (0.04) | 1.07*a*  (0.03) | 1.02*a*  (0.04) | 0.97*a*  (0.03) | 0.84*c*  (0.04) | 0.98*a*  (0.05) |
|  | ***Total*** | **SOM** | 1.05*A*  (0.03) | 0.98*B*  (0.02) | 0.98*A*  (0.03) | 1.01*A*  (0.02) | 1.12*A*  (0.03) | 1.05*A*  (0.04) |
|  |  | **Soil** | 0.03*B*  (0.02) | 1.06*A*  (0.02) | 0.98*A*  (0.02) | 0.99*A*  (0.02) | 0.87*B*  (0.02) | 1.02*A*  (0.03) |

Means with the same letter indicate no significant difference (1) within organic, mineral, mixed fertilizations or (2) within total fertilization according to Tukey’s multiple range test (*P = 0.05*).

**Table S4.** Comparisons of the effects of either composts or raw materials including sewage sludge and manure as organic fertilizers on various functional C groups (*i.e*., Alkyl, O-alkyl, Aromatic, Carbonyl groups) and the ratios of alkyl to O-alkyl (A/O) and alkyl to aromatic (A/Aroma) groups compared to that of the control (*i.e.,* unfertilized system). Numbers in parenthesis are standard errors.

|  | | **Alkyl** | **O-alkyl** | **Aromatic** | **Carbonyl** | **A/O** | **A/Aroma** |
| --- | --- | --- | --- | --- | --- | --- | --- |
| **Organic fertilization** | ***Compost*** | 1.01*a*  (0.06) | 1.03*a*  (0.06) | 0.94*a*  (0.06) | 1.05*a*  (0.06) | 0.87*a*  (0.07) | 1.08*a*  (0.06) |
|  | ***Raw*** | 0.91*a*  (0.08) | 1.09*a*  (0.07) | 0.95*a*  (0.06) | 0.99*a*  (0.06) | 0.81*a*  (0.08) | 0.94*a*  (0.08) |

Means with the same letter indicate no significant difference within the same functional groups according to Tukey’s multiple range test (*P = 0.05*).

**Table S5.** Comparisons of the effects of either animal manures or straw or green waste materials as organic fertilizers on various functional C groups (*i.e*., Alkyl, O-alkyl, Aromatic, Carbonyl groups) and the ratios of alkyl to O-alkyl (A/O) and alkyl to aromatic (A/Aroma) groups compared to that of the control (*i.e.,* unfertilized system). Numbers in parenthesis are standard errors.

|  | | **Alkyl** | **O-alkyl** | **Aromatic** | **Carbonyl** | **A/O** | **A/Aroma** |
| --- | --- | --- | --- | --- | --- | --- | --- |
| **Organic fertilization** | ***Animal manures*** | *0.91a*  (0.06) | 1.05*a*  (0.06) | 0.93*a*  (0.06) | 1.05*a*  (0.06) | 0.88*a*  (0.06) | 1.09*a*  (0.06) |
|  | ***Straw and green waste*** | 0.90*a*  (0.08) | 1.03*a*  (0.08) | 1.10*a*  (0.08) | 0.95*a*  (0.09) | 0.88*a*  (0.08) | 0.96*a*  (0.08) |

Means with the same letter indicate no significant difference within the same functional groups according to Tukey’s multiple range test (*P = 0.05*).
